# Alcohols as inhibitors of ammonia oxidizing archaea and bacteria

**DOI:** 10.1093/femsle/fnad093

**Published:** 2023-09-12

**Authors:** Barbora Oudova-Rivera, Andrew T Crombie, J Colin Murrell, Laura E Lehtovirta-Morley

**Affiliations:** School of Biological Sciences, University of East Anglia, Norwich NR4 7TJ, United Kingdom; School of Biological Sciences, University of East Anglia, Norwich NR4 7TJ, United Kingdom; School of Environmental Sciences, University of East Anglia, Norwich NR4 7TJ, United Kingdom; School of Environmental Sciences, University of East Anglia, Norwich NR4 7TJ, United Kingdom; School of Biological Sciences, University of East Anglia, Norwich NR4 7TJ, United Kingdom

**Keywords:** ammonia oxidizing microorganisms, ammonia monooxygenase, alcohols, inhibition, substrate analogues

## Abstract

Ammonia oxidizers are key players in the global nitrogen cycle and are responsible for the oxidation of ammonia to nitrite, which is further oxidized to nitrate by other microorganisms. Their activity can lead to adverse effects on some human-impacted environments, including water pollution through leaching of nitrate and emissions of the greenhouse gas nitrous oxide (N_2_O). Ammonia monooxygenase (AMO) is the key enzyme in microbial ammonia oxidation and shared by all groups of aerobic ammonia oxidizers. The AMO has not been purified in an active form, and much of what is known about its potential structure and function comes from studies on its interactions with inhibitors. The archaeal AMO is less well studied as ammonia oxidizing archaea were discovered much more recently than their bacterial counterparts. The inhibition of ammonia oxidation by aliphatic alcohols (C_1_-C_8_) using the model terrestrial ammonia oxidizing archaeon ‘*Candidatus* Nitrosocosmicus franklandus’ C13 and the ammonia oxidizing bacterium *Nitrosomonas europaea* was examined in order to expand knowledge about the range of inhibitors of ammonia oxidizers. Methanol was the most potent specific inhibitor of the AMO in both ammonia oxidizers, with half-maximal inhibitory concentrations (IC_50_) of 0.19 and 0.31 mM, respectively. The inhibition was AMO-specific in ‘*Ca*. N. franklandus’ C13 in the presence of C_1_-C_2_ alcohols, and in *N. europaea* in the presence of C_1_-C_3_ alcohols. Higher chain-length alcohols caused non-specific inhibition and also inhibited hydroxylamine oxidation. Ethanol was tolerated by ‘*Ca*. N. franklandus’ C13 at a higher threshold concentration than other chain-length alcohols, with 80 mM ethanol being required for complete inhibition of ammonia oxidation.

## Teaser

Short-chain alcohols specifically inhibit the archaeal and bacterial ammonia monooxygenase, a key enzyme in nitrification, whereas higher chain-length alcohols are non-specific inhibitors of the model ammonia oxidizers ‘*Candidatus* Nitrosocosmicus franklandus’ and *Nitrosomonas europaea*.

## Introduction

Ammonia oxidizing archaea (AOA) and bacteria (AOB) are major microbial drivers of the terrestrial nitrogen cycle, along with comammox bacteria (reviewed in Lehtovirta-Morley 2018). AOA and AOB oxidize ammonia to nitrite, which is further oxidized to nitrate by nitrite oxidizing bacteria. Nitrate is a water pollutant and can be leached from soils, causing eutrophication of aquatic ecosystems. Nitrous oxide is a powerful greenhouse gas and the majority of its anthropogenic emissions are from agricultural soils (Reay et al. 2012). The activity of ammonia oxidizing microorganisms is responsible for significant loss of nitrogen from soil and has broad ecological and economic consequences (Raun and Johnson 1999).

Ammonia monooxygenase (AMO) is a key enzyme in ammonia oxidation and belongs to the copper monooxygenase family (CuMMO). It catalyzes the first step in nitrification, the oxidation of ammonia to hydroxylamine. This enzyme is found in all groups of aerobic ammonia oxidizers. It has never been purified in its active form, which makes this enzyme challenging to study. Current knowledge of the structure and function of this enzyme comes from the similarity of bacterial AMO with the particulate methane monooxygenase (pMMO) from methanotrophs (Lontoh et al. 2000; Semrau et al. 2010; Ross et al. 2019, Zhu et al. 2022), as well as studies based on its interaction with inhibitors (Taylor et al. 2013, 2015, Wright et al. 2020). The archaeal AMO is phylogenetically distinct from the bacterial AMO and pMMO, and its structure is likely to be different (Hodgskiss et al. 2023). Studies comparing the effects of inhibitors, particularly compounds that are substrate analogues, on the archaeal and bacterial AMO can provide insights into the substrate range and function of the AMO.

Bacterial AMO, like other CuMMO superfamily enzymes, can oxidize a wide variety of substrates, including methane, methanol, linear 1-alkanes, alkenes, halogenated hydrocarbons, sulphides, and aromatic compounds (Hyman et al. 1988a; Rasche et al. 1991, Juliette et al. 1993, Keener and Arp 1993, 1994), but oxidation of these substrates does not yield energy and a source of reductant is required for these co-oxidation reactions. There are no known alternative substrates for the archaeal AMO, but several inhibitors have been described. Previous studies found that archaeal AMO from several genera of AOA was inhibited by C_2_ to C_5_ 1-alkynes, and AMO from the bacterium *Nitrosomonas europaea* was inhibited by C_2_-C_8_ 1-alkynes (Taylor et al. 2013, 2015, Wright et al. 2020). This indicates that AOB has a broader substrate and inhibitor range than AOA, and 1-octyne is therefore routinely used for selective inhibition of bacterial (but not archaeal) ammonia oxidizers (Lu et al. 2015, Hink et al. 2017, Lin et al. 2021). Interestingly, phenylacetylene and octyne do not interact with the same site of the archaeal AMO as ammonia and acetylene, and AOA are inhibited by phenylacetylene and octyne at higher concentrations than AOB (Wright et al. 2020). Methane is a competitive inhibitor of the AMO in both archaea and bacteria (Suzuki et al. 1976, Oudova-Rivera et al. 2023). Oxidation of alkanes and alkenes by CuMMO superfamily monooxygenases, including the bacterial AMO, generates alcohols as reaction products (Hyman et al. 1988b). For example, *N. europaea* oxidizes alkanes of up to C_8_, and the reaction products are a mixture of different isomers of alcohols (Hyman et al. 1988b). Whilst these alcohols may be further oxidized by alcohol dehydrogenases in microbial cells, they can also interact with the CuMMO due to their structural similarity to the native substrates. Methanol is also oxidized by bacterial AMO, as was demonstrated by the inhibition of methanol oxidation in *N. europaea* by acetylene or allylthiourea (ATU), specific inhibitors of AMO (Voysey and Wood 1987). Although the production of alcohols by both bacterial AMO and pMMO has been studied previously, very little is known about the inhibition of archaeal and bacterial AMO by alcohols. Recently, methanol was shown to be a specific competitive inhibitor of the archaeal AMO from ‘*Ca*. N. franklandus’ C13 (Oudova-Rivera et al. 2023). Furthermore, specific inhibition of the bacterial AMO from *N. europaea* by short-chain linear C_1_-C_4_ alcohols, including methanol, ethanol, and both isomers of propanol and butanol, was previously reported (Hooper and Terry 1973). Soluble methane monooxygenase (sMMO) from *Methylococcus capsulatus* can oxidize methanol with K_m(app)_ of 0.95 mM (Colby et al. 1977). Constitutive expression of the sMMO in a methanol dehydrogenase double mutant of the facultative methanotroph *Methylocella silvestris* BL2 enables it to grow on methanol and ethanol, suggesting that sMMO can oxidize these alcohols (Crombie 2022).

To our knowledge, the effects of longer-chain alcohols (>C_5_) on either AOA or AOB have not been studied. AOB are sensitive to a wider range of alkynes than AOA, including chain lengths of up to C_9_ (Taylor et al. 2013). Furthermore, AOB are inhibited by octyne and phenylacetylene at a lower concentration than AOA (Taylor et al. 2013, 2015, Wright et al. 2020). It seems plausible, although untested, that responses of AOB and AOA to alcohols may follow similar inhibition patterns. However, the chemical properties of alcohols and alkynes are different, as alcohols contain a polar, hydrophilic hydroxyl group (-OH). The active site of the AMO is thought to reside in a hydrophobic cavity, as it is in pMMO (Ng et al. 2008), and the increasingly amphiphilic nature of longer chain-length alcohols may cause them to interact with membranes differently than alkynes would.

In addition to being potentially useful compounds to study the function of archaeal and bacterial AMO, alcohols naturally occur in soils as products of plant and microbial metabolism. The main sources of methanol, ethanol, and other alcohols in soils include the breakdown of pectin and other methoxylated compounds from plants (Kimmerer and MacDonald 1987, Kirstine and Galbally 2012), root exudation (Smucker and Erickson 1987) and decay of organic matter (Warneke et al. 1999, Isidorov et al. 2003). In addition, methylotrophs are associated with plant roots, suggesting the presence of methanol in the rhizosphere (Macey et al. 2020). AOA and AOB are commonly present in the soil and rhizosphere, and could therefore be exposed to alcohols of various chain lengths. Although there is limited information on the environmental concentrations of different alcohols, their effect may be relevant in specific environmental niches. The aim of this study was to examine the effects of alcohols on model terrestrial ammonia oxidizing archaeon ‘*Ca*. N. franklandus’ C13 and ammonia oxidizing bacterium *N. europaea* using a range of concentrations and chain lengths (C_1_-C_8_).

## Materials and methods

### Materials

Methanol, 1-propanol, 1-hexanol, and 1-heptanol were purchased from Sigma–Aldrich, ethanol and 2-propanol from VWR, 1-butanol and 1-pentanol from Thermo Fisher Scientific, and 1-octanol from Fluka. All alcohols were >99% purity.

### Culture maintenance

Pure cultures of *N. europaea* and ‘*Ca*. N. franklandus’ C13 were grown in freshwater medium (FWM) (pH 7.3), containing 1 g NaCl, 0.4 g MgCl_2_·6H_2_O, 0.1 g CaCl_2_·2H_2_O, 0.2 g KH_2_PO_4_, 0.5 g KCl per litre of ddH_2_O. Medium was supplemented with 5 mM NH_4_Cl, 2 mM NaHCO_3_, 10 mM HEPES, 1 ml l^−1^ modified non-chelated trace element mixture (Könneke et al. 2005), 7.5 µM FeNaEDTA (Tourna et al. 2011), 1 ml l^-1^ vitamin solution (Lehtovirta-Morley et al. 2016). Both *N. europaea* and ‘*Ca*. N. franklandus’ C13 were incubated without shaking at 28°C and 37°C, respectively (Lehtovirta-Morley et al. 2016).

### Whole-cell activity assays for examining inhibition by alcohols

*Nitrosomonas europaea* and ‘*Ca*. N. franklandus’ C13 cells were harvested at mid-exponential phase (∼800 µM NO_2_^−^ accumulated) by filtration onto 0.22 µm pore-size membrane filter (PES, Merck Millipore). Cells were then washed and resuspended in FWM salts (i.e. FWM without added NH_4_Cl and other medium components as described above) buffered with 10 mM HEPES buffer (pH 7). Cells were incubated at 28°C for *N. europaea* and 37°C for ‘*Ca*. N. franklandus’ C13 for ∼1 h until endogenous respiration ceased.

A total volume of 5 ml of cell suspension was placed in 24 ml glass vials, alcohols (C_1_-C_8_) were added to final concentrations in the range of 2–100 mM and immediately sealed with twice-autoclaved gray butyl rubber stoppers and crimp sealed. Hexanol, heptanol, and octanol have relatively low solubility in water, reaching full saturation at 58, 17, and 2 mM, respectively. Although these alcohols were added at concentrations above their solubility for the consistency of the experimental design, the limit of solubility was considered when evaluating the results. Reactions were initiated by addition of 500 µM NH_4_Cl or 200 µM NH_2_OH. Vials containing *N. europaea* and ‘*Ca*. Nitrosocosmicus franklandus’ C13 cells were incubated at 28°C and 37°C, respectively. The activity of cultures was followed by monitoring nitrite accumulation over 1 h, measured in 15 min intervals using Griess reagent in a 96-well plate format as previously described (Lehtovirta-Morley et al. 2014). The final cell concentration in the activity assays was ∼10^7^ cells ml^−1^ for both *N. europaea* and ‘*Ca*. N. franklandus’ C13, which equate to ∼1.2 and 5.55 µg protein ml^−1^, respectively. Cell counts were performed as previously described (Oudova-Rivera et al. 2023). Protein concentrations were determined using a Pierce bicinchoninic acid (BCA) protein assay kit (Thermo Scientific) according to the manufacturer instructions. One-way ANOVA with LSD post-hoc tests were performed using SPSS statistics software version 27 (IBM).

The reaction velocity of ammonia and hydroxylamine oxidation was determined by measuring the rate of nitrite accumulation over time (µM min^−1^). The logarithm of the reaction velocity, calculated as a percentage of the non-inhibited control, was plotted against the inhibitor concentration resulting in a linear relationship (Figs. S1 and S2). The half-maximal inhibitory concentration (IC_50_) was calculated from the slope and y-axis intercept over the linear part of the curve following the formula:


\begin{equation*}
IC50 = \frac{{{\mathrm{log}}\left( {50} \right) - b}}{m},
\end{equation*}

where *b* is the estimated y-axis intercept and *m* is the estimated slope.

Inhibition by alcohols was tested at a fixed substrate concentration of 500 µM NH_4_Cl or 200 µM hydroxylamine for both *N. europaea* and ‘*Ca*. N. franklandus’ C13. Ammonia and hydroxylamine concentrations were selected based on previously published K_m_ values and activity assays (Suzuki et al. 1974, Martens-Habbena et al. 2009, Oudova-Rivera et al. 2023). Fully inhibitory concentrations of alcohols were determined experimentally with a series of activity assays, gradually narrowing down the range of alcohol concentrations applied. Full inhibition was defined as a lack of sustained nitrite accumulation, where there was either no nitrite accumulation at all, or where a minor accumulation of nitrite was initially detected but ceased (potentially due to a delayed effect of the inhibitor).

## Results and discussion

### Inhibition of the ammonia oxidizing archaeon ‘*Ca*. N. franklandus’ C13 by alcohols

Inhibition of ammonia oxidation was tested using whole-cell activity assays with the concentration of alcohols ranging from 2  to 100 mM and fixed substrate ammonia concentration of 500 µM NH_4_Cl. Specificity of the inhibition of AMO was tested by activity assays with 200 µM hydroxylamine and a concentration of each alcohol that resulted in complete inhibition of ammonia oxidation when using ammonia as substrate (Table 1). Because hydroxylamine, the product of ammonia oxidation, is a pathway intermediate downstream of AMO, any inhibitors specific to AMO should not interfere with hydroxylamine oxidation.

**Table 1.** Half-maximal inhibitory concentrations (IC_50_) and lowest fully inhibitory concentrations* of alcohols (I) in ‘*Ca*. N. franklandus’ and *N. europaea*

	‘*Ca*. N. franklandus’		*N. europaea*	
	IC_50_ [mM]	I [mM]	IC_50_ [mM]	I [mM]
Methanol	0.19	8	0.31	10
Ethanol	7.2	80	20.9	90
1-Propanol	6.2	20	13.6	100
2-Propanol	26.8	40	32.6	100
Butanol	4.0	40	7.6	100
Pentanol	3.6	20	5.3	100
Hexanol	2.4	15	2.2	20
Heptanol	3.6	17	4.5	17
Octanol	N/A**	10**	N/A**	20**

* Full inhibition was defined as a lack of sustained nitrite accumulation, where there was either no nitrite accumulation at all, or where a minor accumulation of nitrite was initially detected but ceased (potentially due to a delayed effect of the inhibitor).

** The limit of solubility for octanol in water is 2 mM.

**Figure 1.**
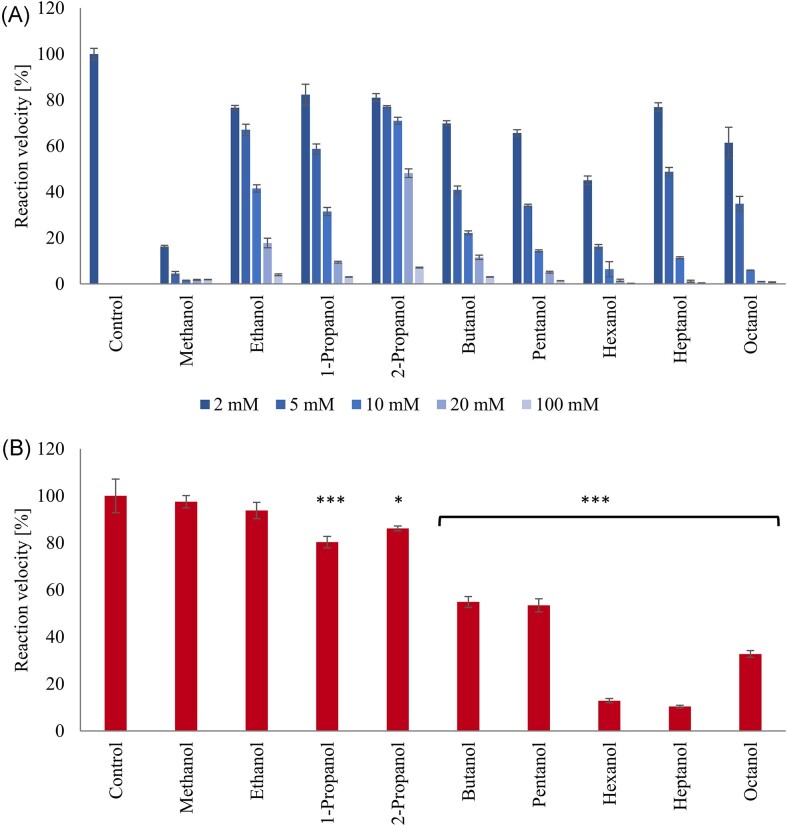
Inhibition of nitrite production by *‘Ca*. N. franklandus’ (AOA) in response to linear alcohols in concentration ranging from 2 to 100 mM and supplied with 500 µM NH_4_Cl (panel **A**), and in response to the lowest fully inhibitory concentration of each alcohol and supplied with 200 µM hydroxylamine (panel **B**). The reaction velocities of the non-inhibited controls were 177 nmol NO_2_^−^ mg protein^−1 ^min^−1^ and 52 nmol NO_2_^−^ mg protein^−1 ^min^−1^ for ammonia and hydroxylamine as substrates, respectively. Significant differences between cells inhibited with the alcohols shown and non-inhibited controls are shown as *P < 0.05; *** 10 *P* < 0.001. Error bars represent the standard error (*n* = 3).

Both methanol and ethanol were specific inhibitors of the archaeal AMO in ‘*Ca*. N. franklandus’ C13 and did not interfere with hydroxylamine oxidation (Fig. 1). However, hydroxylamine oxidation was increasingly inhibited by the increasing chain lengths of alcohols of > C_3_. Complete inhibition of ammonia oxidation was achieved at 8 mM methanol and the IC_50[methanol]_ was 0.19 mM (Table 1), making methanol the most potent inhibitor tested. Nitrite accumulation was not affected when the cells were supplied with hydroxylamine and 8 mM methanol, indicating that methanol is a specific inhibitor of the AMO (Fig. 1) as previously reported (Oudova-Rivera et al. 2023). In contrast to methanol, ‘*Ca*. N. franklandus’ C13 tolerated relatively high concentrations of ethanol. A total concentration of 80 mM ethanol was required for complete inhibition of ammonia oxidation in ‘*Ca*. N. franklandus’ C13 and the IC_50_ for ethanol (7.2 mM) was more than an order of magnitude higher than the IC_50_ for methanol. Although our study focussed on the effect of primary alcohols on ammonia oxidizers, a secondary alcohol (2-propanol) was also included. ‘*Ca*. N. franklandus’ C13 tolerated 2-propanol better than 1-propanol, with IC_50_ values 26.8 and 6.2 mM, respectively (Table 1).

In ‘*Ca*. N. franklandus’ C13, the inhibition by longer chain linear alcohols (>C_4_) was not specific to AMO, as they inhibited the oxidation of hydroxylamine as well as oxidation of ammonia (Fig. 1). At butanol and pentanol concentrations fully inhibitory to ammonia oxidation, the rates of nitrite accumulation from hydroxylamine oxidation were 55% ±2.3% and 53% ± 2.8%, respectively, compared to the non-inhibited control. A total volume of 15 mM hexanol completely inhibited ammonia oxidation with a IC_50[hexanol]_ of 2.4 mM (Table 1), and the nitrite accumulation rate in the presence of hydroxylamine was only 13% ± 0.9% compared to the non-inhibited control. Similarly, heptanol caused full inhibition at 17 mM concentration, IC_50[heptanol]_ was 3.6 mM and providing hydroxylamine as a substrate resulted in 10% ± 0.5% activity compared to non-inhibited control. The limit of solubility for octanol in water is 2 mM, a concentration which inhibited ammonia oxidation by 39%. Additional octanol caused further inhibition, possibly because of its general toxicity. Full inhibition was achieved when 10 mM octanol was added. However, 33% ± 1.4% of oxidation activity was restored in the presence of hydroxylamine.

### Inhibition of the ammonia oxidizing bacterium *N. europaea* by alcohols

**Figure 2.**
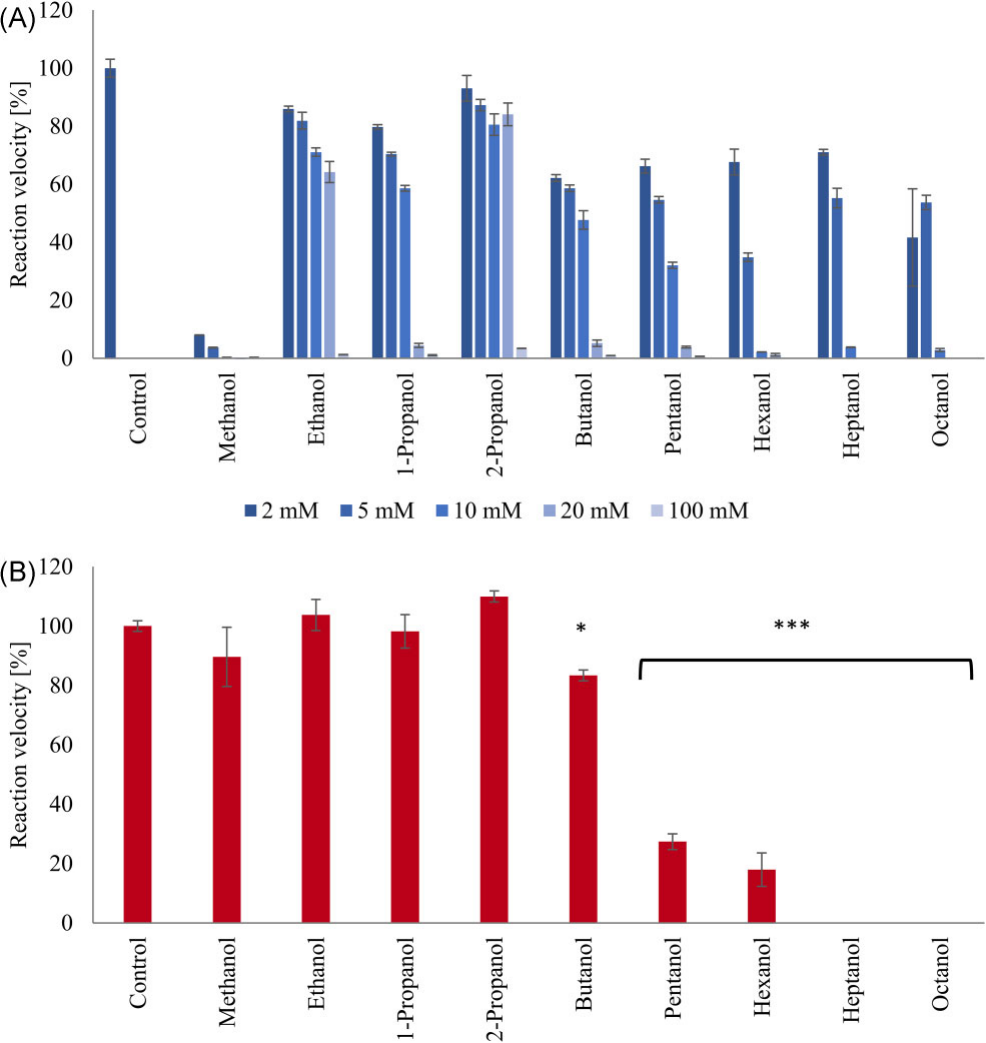
Inhibition of nitrite production by *N. europaea* (AOB) in response to linear alcohols in concentration ranging from 2 to 100 mM and supplied with 500 µM NH_4_Cl (panel **A**), and in response to the lowest fully inhibitory concentration of each alcohol and supplied with 200 µM hydroxylamine (panel **B**). The reaction velocities of the non-inhibited controls were 1.26 µmol NO_2_^−^ mg protein^−1 ^min^−1^ and 0.54 µmol NO_2_^−^ mg protein^−1 ^min^−1^ for ammonia and hydroxylamine as substrates, respectively. Significant differences between cells inhibited with the alcohols shown and non-inhibited controls are shown as *P < 0.05; ***P < 0.001. Error bars represent standard error (*n* = 3).

As observed for the AOA ‘*Ca*. N. franklandus’ C13, the most potent inhibitor of *N. europaea* among all alcohols tested was methanol (IC_50[methanol]_ = 0.31 mM) (Fig. 2, Table 1). As with methanol, ethanol was a specific inhibitor of both archaeal and bacterial AMO. Consistent with the findings in ‘*Ca*. N. franklandus’ C13, *N. europaea* tolerated relatively high concentrations of ethanol with 90 mM ethanol required for complete inhibition of ammonia oxidation. The IC_50_ for ethanol in both microorganisms was one to two orders of magnitude greater than for methanol (7.2 and 20.9 mM for ‘*Ca*. N. franklandus’ C13 and *N. europaea*, respectively). The cause for the substantial difference between the inhibition profiles of methanol and ethanol is not clear but may be related to production of aldehydes, which are potential oxidation products of alcohols, or to free radical scavenging, as suggested by Hooper and Terry (1973).

Unlike with ‘*Ca*. N. franklandus’ C13, with *N. europaea* both isomers of propanol were specific inhibitors of AMO (Fig. 2B). 2-propanol was better tolerated than 1-propanol, with the highest IC_50_ (32.6 mM) for 2-propanol. This differs from results obtained by Hooper and Terry (1973), who found that *N. europaea* was maximally inhibited by a lower concentration of 2-propanol (0.13 M) than 1-propanol (0.33 M). Observed differences between the isomers of propanol are unlikely to be due to steric hindrance alone, because hydrocarbons of <C_5_ are thought to interact with the active site of the bacterial and archaeal AMOs (Taylor et al. 2013, Wright et al. 2020).

Longer chain-length alcohols (>C_4_) were non-specific inhibitors of ammonia oxidation by *N. europaea*. Butanol inhibited nitrite accumulation from ammonia with a IC_50_ of 7.6 mM. In the presence of hydroxylamine at fully inhibitory butanol concentration, the reaction velocity was significantly reduced to 83% ± 1.8% compared to the control, suggesting that AMO was not the sole target of inhibition. Pentanol (IC_50_ = 5.3 mM) and hexanol (IC_50_  = 2.2 mM) applied at a concentration which was fully inhibitory in the presence of ammonia, only allowed for minor accumulation of nitrite when cells were supplied with hydroxylamine, at rates of 27% ± 2.6% and 18% ± 5.6%, respectively, compared to the control. The inhibition of ammonia oxidation by heptanol (IC _50_ = 4.5 mM) as with octanol was non-specific, with no activity detected in the presence of hydroxylamine.

### Significance of findings in the context of ecology and physiology of ammonia oxidation

This study compared the inhibition by aliphatic alcohols of two widespread model nitrifiers, the archaeon ‘*Ca*. N. franklandus’ C13 and the bacterium *N. europaea*. Methanol and ethanol were specific inhibitors of the AMO, and longer-chain alcohols (C_4_-C_8_) were non-specific inhibitors of both nitrifiers. With the exception of ethanol, *N. europaea* was more tolerant than ‘*Ca*. N. franklandus’ C13 to all alcohols tested. Although there is currently a lack of knowledge about environmental sources, sinks and concentrations of alcohols, especially longer-chain alcohols (>C_5_), a few studies have measured concentrations of methanol, ethanol, and propanol predominantly in aquatic habitats. Methanol concentrations in the seawater range between 61 and 97 nM (Dixon et al. 2011, Bates et al. 2021). In the Atlantic, the concentration of ethanol was reported to be 2–33 nM, and concentrations of 1-propanol and 2-propanol 2–22 nM and 1–19 nM, respectively (Beale et al. 2010). In an ice core from Greenland, methanol concentrations were 83–1666 nM and ethanol 16–424 nM (Felix et al. 2019). In soil ecosystems, methanol and ethanol are present in the rhizosphere as a component of root exudates or product of decomposition and fermentation (Young et al. 1977, Haldar and Sengupta 2015, Macey et al. 2020). Concentrations of ethanol in the rhizosphere of *Lupinus angustifolius* L. were found to be between 1 and 5 mM (Young et al. 1977), which is significantly higher than in e.g. seawater and could potentially have minor inhibitory effect on ammonia oxidizers. Although methanol concentration in soil is unknown, it has been suggested that there may be ‘hotspots’ with elevated methanol concentration in the vicinity of plant material and soil methylotrophs have been shown to oxidize methanol at low (<10 µM) concentrations (Stacheter et al. 2013). In addition, it is estimated that 28×10^12^ mol yr^−1^ of methanol are produced from decaying and living plant material, but only 4.9×10^12^ mol yr^−1^ of methanol are emitted from soils, potentially suggesting that the gap between production and emission of methanol in soils may be due to active methanol oxidizing microbial communities (Kolb 2009). Both AOA and AOB are ubiquitous microorganisms, and AOA can sometimes dominate the archaeal community in the rhizosphere (Prudence et al. 2021). Intriguingly, a previous DNA stable isotope probing study using ^13^C-labelled methanol in soil microcosms retrieved a metagenome-assembled genome affiliated to the genus *Candidatus* Nitrosocosmicus in the ^13^C-DNA fraction, implying that methanol-derived carbon was either directly or indirectly assimilated by soil AOA (Macey et al. 2020). Nevertheless, the question as to whether aliphatic alcohols could affect nitrification in soil ecosystems remains largely unexplored.

The inhibition of both archaeal and bacterial AMO by linear alcohols was very different than the results previously reported on inhibition by alkynes. The inhibitory effect of alkynes is weaker with increasing chain length in AOA, whereas *N. europaea* is also inhibited by long-chain alkynes (Taylor et al. 2013, Wright et al. 2020). In contrast to alkynes, there was no substantial difference between the inhibition profiles of AOA and AOB with increasing chain lengths of alcohols. In addition, the inhibition of ammonia oxidizers by alkynes is specific to the AMO, whereas alcohols >C_3_ also inhibited hydroxylamine oxidation in both AOA and AOB. Alcohols contain a hydrophilic hydroxyl group absent in alkynes. Although the active site of the AMO is thought to be hydrophobic (Welton and Reichardt 2011), polarity alone is unlikely to explain the differences observed. Alcohols are solvents and may interact with cellular structure, induce solvent stress and influence fluidity and permeability of membranes. Thaumarchaeal membranes consist of glycerol dibiphytanyl glycerol tetraether (GDGT) lipids, in contrast to the phospholipid bilayer found in bacteria (Bale et al. 2019). Interestingly, a previous study found that archaea from phylum Euryarchaeota were more sensitive to alcohol stress than bacteria (Huffer et al. 2011). Although the membrane composition of members of the phylum Euryarchaeota is different to that of Thaumarchaeota (Villanueva et al. 2014), the greater sensitivity to alcohols compared to bacteria appears to be in line with these findings.

This study provides insights into the inhibition of ammonia oxidation by alcohols in the model nitrifiers archaeon ‘*Ca*. N. franklandus’ C13 and bacterium *N. europaea*. This work contributes to understanding of the function of AMO, but also raises questions about whether alcohols could affect nitrification under environmentally relevant conditions and whether other strains of AOA, AOB, and comammox *Nitrospira* would respond in a manner similar to the model organisms used in our study. Future studies should explore these questions and investigate the roles of cell envelope and membranes of ammonia oxidizers in withstanding stressors such as alcohols.

## Supplementary Material

fnad093_Supplemental_File
