# Supplementary material for: Alcohols as inhibitors of ammonia oxidizing archaea and bacteria
Source: FEMS Microbiol Lett. 2023 Sep 12;370:fnad093. doi: 10.1093/femsle/fnad093 (PMC11025371; doi:10.1093/femsle/fnad093)
Supplement: fnad093_Supplemental_File [file fnad093_supplemental_file.docx]

**Supplementary material**


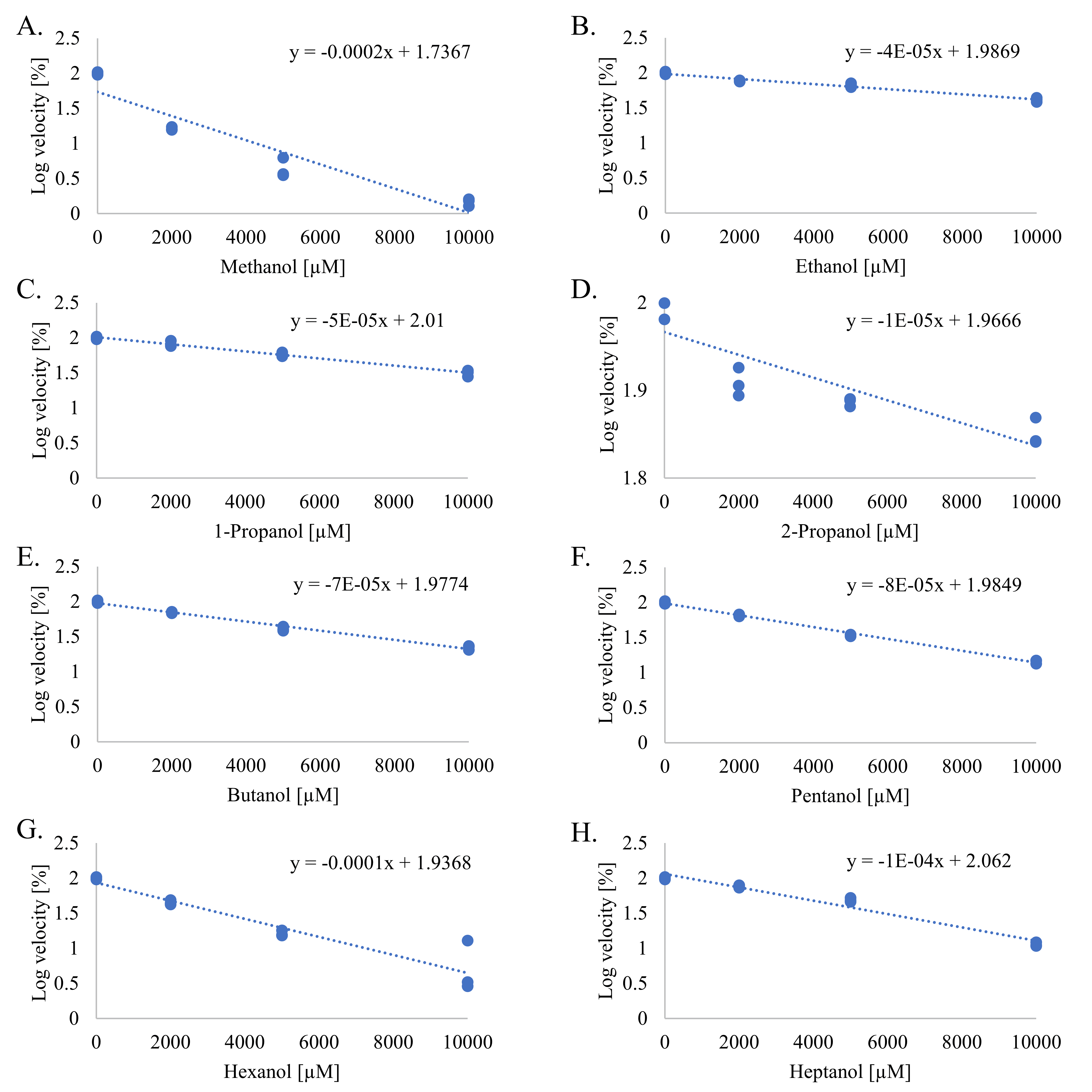


**Supplementary Figure 1.** Linear regression plots of inhibitor concentrations vs. logarithm of the reaction velocity (as a percentage of the non-inhibited control) for ammonia oxidation in ‘Ca. N. franklandus’ C13 (n=3). A. Methanol, B. Ethanol, C. 1-Propanol, D. 2-Propanol, E. Butanol, F. Pentanol, G. Hexanol, H. Heptanol.


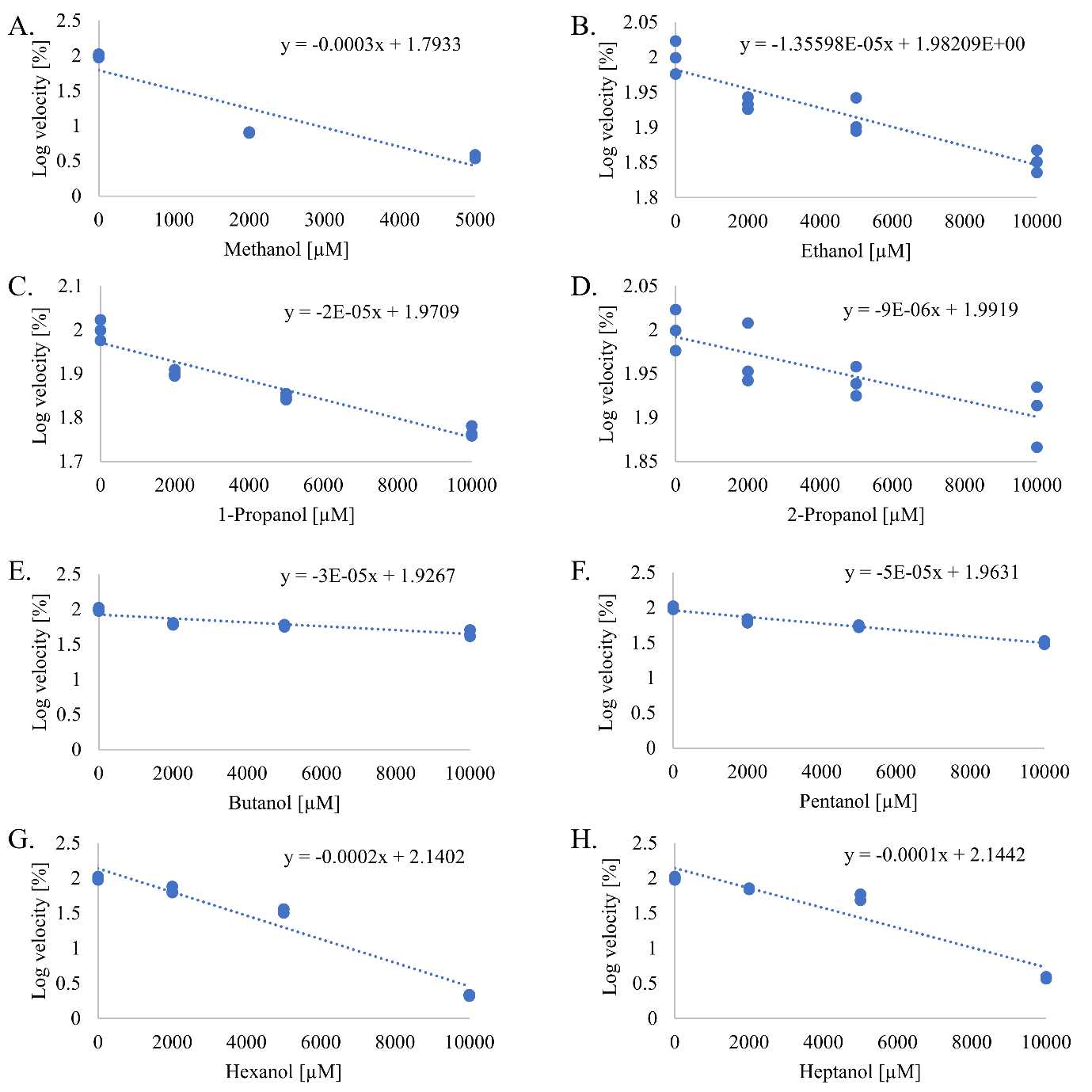


**Supplementary Figure 2.** Linear regression plots of inhibitor concentrations vs. logarithm of the reaction velocity (as a percentage of the non-inhibited control) for ammonia oxidation in N. europaea (n=3). A. Methanol, B. Ethanol, C. 1-Propanol, D. 2-Propanol, E. Butanol, F. Pentanol, G. Hexanol, H. Heptanol.
